# The role of nutritional status and sarcopenia in treatment tolerance and toxicity in head and neck cancer: a narrative review

**DOI:** 10.3389/fnut.2026.1924291

**Published:** 2026-08-28

**Authors:** Ana Cebrián-Vázquez, Agustín Ramos-Prol, Erika Vieira-Maroun, María Argente-Pla, Juan Francisco Merino-Torres

**Affiliations:** 1 Endocrinology and Nutrition Department, La Fe University and Polytechnic Hospital in Valencia, Valencia, Spain; 2 Joint Research Unit on Endocrinology, Nutrition and Clinical Dietetics, Health Research Institute La Fe, Valencia, Spain; 3 Department of Medicine, Faculty of Medicine, University of Valencia, Valencia, Spain

**Keywords:** body composition, head and neck cancer, malnutrition, morphofunctional assessment, muscle ultrasound, prehabilitation, sarcopenia, treatment toxicity

## Abstract

**Introduction:**

Malnutrition, sarcopenia, and adverse body-composition phenotypes are highly prevalent in patients with head and neck cancer (HNC) and have been increasingly recognized as clinically relevant predictors of treatment tolerance, treatment-related toxicity, and oncologic outcomes. Morphofunctional assessment has emerged as a comprehensive approach for evaluating nutritional and functional status beyond conventional anthropometric measures.

**Methods:**

A narrative review was conducted using a structured literature search in PubMed/MEDLINE, Scopus, and Web of Science from database inception to June 2026. Studies evaluating the relationship between nutritional status, sarcopenia, body composition, treatment tolerance, treatment-related toxicity, functional outcomes, and prognosis in adult patients with HNC were considered. Clinical practice guidelines, systematic reviews, meta-analyses, and original studies published in English or Spanish were included.

**Results:**

Current evidence demonstrates that malnutrition, sarcopenia, and low skeletal muscle mass are consistently associated with increased treatment-related toxicity, poorer treatment tolerance, prolonged hospitalization, higher postoperative complication rates, and reduced survival. Body-composition assessment, particularly through computed tomography–based analysis, provides clinically relevant prognostic information beyond conventional anthropometric parameters. Emerging evidence also supports the role of morphofunctional assessment, including muscle strength, physical performance, and muscle ultrasound, for identifying patients at nutritional risk and improving risk stratification. In parallel, multimodal supportive-care strategies integrating nutritional intervention, exercise, and prehabilitation have shown potential to preserve functional status and improve treatment preparedness.

**Conclusion:**

Malnutrition, sarcopenia, and adverse body-composition phenotypes represent important predictors of clinical outcomes in patients with HNC. The incorporation of morphofunctional assessment into routine clinical practice may facilitate earlier identification of vulnerable patients and support more individualized nutritional and rehabilitative interventions. Future research should prioritize longitudinal morphofunctional assessment, standardization of assessment methods, and the integration of personalized supportive-care strategies throughout the oncologic trajectory.

## 1 Introduction

Head and neck cancer (HNC) represents one of the most nutritionally challenging malignancies in oncology. Globally, it is the seventh most common cancer, with more than 900,000 new cases and over 400,000 deaths annually worldwide (1, 2). The vast majority correspond to squamous cell carcinomas arising from the oral cavity, pharynx, and larynx, and are strongly associated with tobacco and alcohol consumption, as well as human papillomavirus (HPV) infection, particularly in oropharyngeal tumours (2, 3). Despite advances in multimodal treatment strategies, many patients are still diagnosed at locally advanced stages, requiring aggressive therapeutic approaches including surgery, radiotherapy, chemotherapy, or combined chemoradiotherapy. These therapies are frequently associated with severe nutritional deterioration, loss of skeletal muscle mass, dysphagia, and significant adverse effects, all of which may compromise treatment tolerance, functional status, quality of life, and oncologic outcomes (4).

Malnutrition is highly prevalent among patients with head and neck malignancies, affecting approximately 35%–60% of patients at diagnosis, with prevalence increasing substantially during treatment (5, 6). The etiology is multifactorial and involves a complex interaction between tumour-induced metabolic alterations, systemic inflammation, reduced oral intake, and therapy-associated toxicities. Dysphagia, odynophagia, xerostomia, mucositis, taste alterations, anorexia, and gastrointestinal symptoms frequently contribute to inadequate nutritional intake and progressive weight loss in this population (6). In parallel, cancer-related inflammatory and catabolic pathways promote loss of skeletal muscle mass, altered protein turnover, insulin resistance, and increased resting energy expenditure, ultimately favouring the development of cachexia and sarcopenia (5, 7). Unlike simple starvation, cancer cachexia is characterized by persistent metabolic dysregulation and ongoing muscle wasting that cannot be fully reversed by conventional nutritional support alone (7).

In recent years, sarcopenia has emerged as a particularly relevant prognostic factor in HNC. Reduced skeletal muscle mass and function have been associated with increased oncologic treatment toxicity, higher rates of treatment interruption, prolonged hospitalization, postoperative complications, and poorer oncologic outcomes (8). Furthermore, sarcopenia may also be present in overweight or obese patients, a condition referred to as sarcopenic obesity, which can remain underrecognized when nutritional assessment relies exclusively on body weight or body mass index (BMI) (5). These findings highlight the importance of comprehensive nutritional and morphofunctional assessment in patients undergoing multimodal oncologic treatment.

Therapy-related toxicity represents one of the major contributors to poorer prognosis and reduced quality of life in patients with HNC. Radiotherapy and chemoradiotherapy are frequently associated with severe acute and chronic adverse effects, including oral mucositis, dysphagia, xerostomia, aspiration, dehydration, weight loss, and long-term swallowing dysfunction (9, 10). These complications may lead to unplanned hospital admissions, enteral feeding dependence, treatment interruptions, and reduced adherence to oncologic therapy, all of which can negatively affect clinical outcomes (10, 11). In this context, growing evidence suggests that baseline malnutrition and sarcopenia are associated with an increased risk of treatment-related toxicities and reduced treatment tolerance (8, 12). Sarcopenic patients have been shown to experience higher rates of chemotherapy dose-limiting toxicity, postoperative complications, prolonged hospitalization, and poorer survival outcomes (8, 13). Moreover, progressive nutritional deterioration during treatment may further exacerbate systemic inflammation and functional decline, perpetuating a vicious cycle that negatively impacts treatment adherence, quality of life, and survival (5, 7).

Given the major clinical implications of malnutrition and sarcopenia in HNC, early identification of patients at nutritional risk is essential. Current guidelines recommend systematic nutritional screening and comprehensive assessment throughout the oncologic trajectory, particularly in patients receiving multimodal treatment (4, 5). However, conventional anthropometric parameters such as body weight or BMI may underestimate clinically relevant alterations in body composition, especially in patients with sarcopenic obesity (5, 8, 14). Consequently, increasing attention has been directed toward morphofunctional assessment tools capable of identifying skeletal muscle depletion and functional impairment at earlier stages. Computed tomography-based body composition analysis has emerged as one of the most widely used methods for assessing skeletal muscle quantity and quality in oncology and has demonstrated substantial prognostic value in patients with HNC (13, 14). In addition, handgrip strength, bioelectrical impedance analysis (BIA), functional performance tests, and muscle ultrasound have gained relevance as complementary tools for the assessment of muscle mass and function in clinical practice (15–17). Muscle ultrasound offers several practical advantages, including bedside applicability, absence of radiation exposure, low cost, and the possibility of longitudinal evaluation of muscle quantity and quality, making it an increasingly attractive tool in nutritional oncology and morphofunctional assessment (17). These approaches may facilitate earlier nutritional interventions and contribute to more individualized supportive care strategies in patients undergoing cancer treatment.

Therefore, understanding the interplay between malnutrition, sarcopenia, body composition changes, and treatment-related toxicity has become essential to optimize supportive care strategies and improve clinical outcomes in patients with HNC. Despite growing evidence, important challenges remain regarding the identification, monitoring, and management of nutritional and morphofunctional alterations in patients with HNC.

The aim of this narrative review is to summarize current evidence regarding the impact of malnutrition and sarcopenia on treatment tolerance, toxicity, and oncologic outcomes in patients with HNC, with particular emphasis on morphofunctional assessment and emerging nutritional and supportive care strategies.

## 2 Methods

This narrative review was conducted to provide an overview of the current evidence regarding the relationship between nutritional status, malnutrition, sarcopenia, body composition, treatment tolerance, and treatment-related toxicity in patients with HNC. Literature searches were conducted in PubMed/MEDLINE, Scopus, and Web of Science from database inception to June 2026.

The search strategy combined terms related to HNC, nutritional and morphofunctional assessment (including malnutrition, cachexia, sarcopenia, body composition, GLIM, computed tomography, bioelectrical impedance analysis, handgrip strength, and muscle ultrasound), and clinically relevant outcomes such as treatment toxicity, treatment tolerance, postoperative complications, hospitalization, functional decline, quality of life, survival, and prognosis. Original studies, clinical practice guidelines, systematic reviews, meta-analyses, and consensus statements published in English or Spanish were considered. Additional relevant publications were identified through manual screening of reference lists of selected articles.

The literature was selected according to its relevance to the objectives of this review, with particular emphasis on studies addressing the relationship between nutritional status, malnutrition, sarcopenia, body composition, or morphofunctional assessment and clinically relevant outcomes in adult patients with HNC. When multiple publications addressed similar topics, priority was given to recent systematic reviews, meta-analyses, clinical practice guidelines, and high-quality prospective studies. Landmark studies introducing key concepts or assessment methodologies were also included when considered essential to provide historical context.

Given the narrative nature of this review, the retrieved evidence was critically interpreted and synthesised according to the major thematic areas addressed in the manuscript. No formal systematic review methodology, predefined study selection protocol, or risk-of-bias assessment was undertaken.

## 3 Nutritional and morphofunctional assessment in head and neck cancer

### 3.1 Pathophysiology of malnutrition and sarcopenia in head and neck cancer

**Figure 1:**
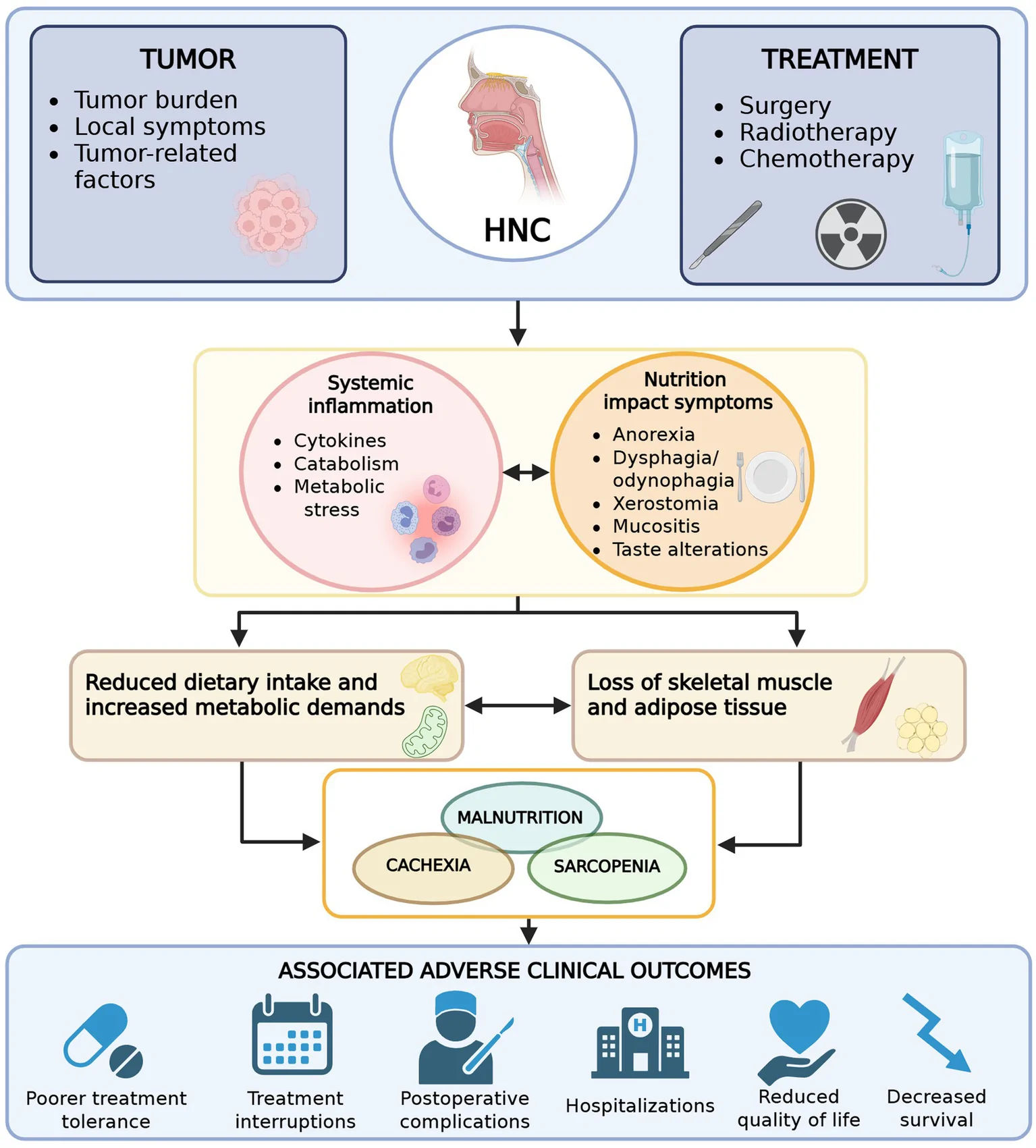
Pathophysiology of malnutrition, sarcopenia and cachexia in patients with head and neck cancer. Created in BioRender. Cebrián Vázquez, A. (2026), https://BioRender.com/6a9ljb5.

Malnutrition and sarcopenia in HNC arise from a multifactorial interplay between tumour-induced systemic metabolic alterations and local factors that impair oral intake. The presence of the tumour and the host response trigger a chronic inflammatory and catabolic state mediated by pro-inflammatory cytokines, including tumour necrosis factor-alpha (TNF-*α*), interleukin-1 (IL-1), and interleukin-6 (IL-6), as well as other tumour and host-derived mediators (7, 18–20). This environment promotes a negative protein-energy balance characterized by systemic inflammation, insulin resistance, disturbances in protein and lipid metabolism and, in a proportion of patients, hypermetabolism and reduced metabolic efficiency (7, 18, 20). At the tissue level, these alterations contribute to progressive skeletal muscle and adipose tissue depletion through activation of catabolic pathways involved in muscle proteolysis and adipose tissue remodelling, including increased ubiquitin-proteasome activity, autophagy, and alterations in adipose tissue metabolism (20). Among the intracellular pathways involved, activation of nuclear factor-kappa B (NF-κB) represents a central molecular mechanism linking inflammation to skeletal muscle catabolism by upregulating components of the ubiquitin–proteasome system and contributing to anabolic resistance and muscle atrophy (21). Consequently, patients experience progressive loss of muscle and fat mass, together with hepatic metabolic adaptations such as increased acute-phase protein synthesis and broader disturbances in energy metabolism. Collectively, these alterations contribute to the development of cancer anorexia-cachexia syndrome and functional decline (7, 18, 20). Figure 1 summarizes the complex pathophysiological mechanisms linking tumour-related factors, systemic inflammation, reduced oral intake, alterations in body composition, and their impact on nutritional status and clinical outcomes in patients with HNC.

Reduced oral intake represents another major driver of nutritional deterioration in patients with HNC, acting synergistically with the systemic inflammatory and catabolic processes described above. Cancer-related anorexia may be mediated by central inflammatory signalling pathways acting on the hypothalamus, while local alterations such as dysgeusia, xerostomia, pain, and oral somatosensory disturbances significantly reduce appetite and impair food intake (7, 20, 22). In addition, the physical presence of the tumour may cause important mechanical and functional alterations that compromise eating and swallowing. Dysphagia frequently develops as a consequence of tumour-related obstruction or functional impairment of structures involved in swallowing (23), whereas odynophagia may result from local inflammation, ulceration, and neural involvement secondary to tumour infiltration. Likewise, impairment of structures responsible for airway protection may increase the risk of aspiration, potentially leading to pneumonia and other respiratory complications (23).

The involvement of anatomical structures essential for mastication, including the masticatory muscles, temporomandibular joint, tongue, and dentition, may result in trismus, reduced masticatory efficiency, and impaired oral food processing (24, 25). Consequently, many patients develop difficulties tolerating regular food textures and progressively shift toward softer, homogeneous, moist, or texture-modified diets (26). These functional limitations contribute to inadequate nutritional intake and progressive nutritional deterioration, which may further exacerbate malnutrition, accelerate functional decline, and negatively affect overall clinical status (20, 26, 27).

Cancer cachexia is a multifactorial syndrome characterized by an ongoing loss of skeletal muscle mass, with or without loss of adipose tissue, that cannot be fully reversed by conventional nutritional support and arises from the combined effects of reduced food intake and tumour-driven metabolic abnormalities (18, 19). Although malnutrition, cachexia, and sarcopenia frequently coexist in patients with HNC, they are not synonymous. In line with current consensus, sarcopenia is defined by low muscle strength together with reduced muscle quantity and/or quality (28), whereas many oncologic studies use the term operationally to denote radiologically defined low skeletal muscle mass. Sarcopenic obesity further complicates recognition because clinically relevant muscle depletion may be masked by preserved or excess adiposity (14, 29). These conceptual distinctions are important when interpreting the literature on treatment tolerance, prognosis, and nutritional assessment in HNC. These mechanisms are not static and frequently intensify during multimodal treatment, leading to further deterioration in nutritional status, body composition, and physical function across the oncologic pathway.

### 3.2 Impact of nutritional status on tolerance to oncologic treatment

Baseline malnutrition, low skeletal muscle mass, and other adverse body-composition phenotypes are increasingly recognized as clinically relevant risk markers of poorer tolerance to oncologic treatment in patients with HNC. Patients who begin treatment with malnutrition, sarcopenia, or low skeletal muscle mass are more likely to experience treatment-related toxicities that further compromise oral intake, functional status, and treatment adherence. Across observational cohorts and meta-analyses, pretreatment sarcopenia (according to consensus definitions) or radiologically defined low skeletal muscle mass has been associated with higher treatment-related toxicity, prolonged hospitalization, treatment interruption, and poorer survival outcomes, although effect estimates vary according to treatment setting, imaging methodology, and the cut-off values used to define sarcopenia (8, 12, 13, 30).

Systemic therapy may further aggravate nutritional vulnerability through anorexia, dysgeusia, oral mucositis, dysphagia, gastrointestinal symptoms, and fatigue (31–34). These toxicities may reduce oral intake, accelerate weight and muscle loss, and increase the need for nutritional support, treatment modification, or dose reduction. Consequently, patients with pre-existing malnutrition or sarcopenia may be particularly susceptible to treatment-related complications and deterioration in nutritional and functional status during therapy (31–34).

Radiotherapy and concurrent chemoradiotherapy are among the treatment modalities associated with the greatest nutritional burden in HNC. Progressive mucositis, dysphagia, xerostomia, taste dysfunction, and aspiration may substantially impair swallowing function and oral intake, frequently necessitating texture-modified diets, oral nutritional supplementation, or enteral feeding support (9, 35–38). In this setting, pretreatment malnutrition and low skeletal muscle mass have been associated with greater treatment-related toxicity, increased supportive-care requirements, poorer treatment completion, and less favourable survival outcomes (8, 9, 12, 13, 30). Furthermore, late toxicities such as fibrosis, osteoradionecrosis, persistent swallowing dysfunction, and fatigue may prolong nutritional vulnerability long after treatment completion (36, 39, 40).

Surgical treatment may also precipitate or exacerbate nutritional compromise, particularly following extensive resections and reconstructive procedures affecting mastication and swallowing. Postoperative dysphagia, temporary or prolonged inability to maintain adequate oral intake, and increased protein-energy requirements associated with wound healing and recovery may further impair nutritional status (41–43). Consistent evidence from systematic reviews and meta-analyses indicates that preoperative sarcopenia and low skeletal muscle mass are associated with a higher risk of postoperative complications, prolonged hospital stays, and less favourable postoperative recovery following head and neck cancer surgery (44–46).

In recent years, proton therapy has emerged as a promising alternative to conventional radiotherapy because of its ability to deliver highly conformal radiation doses while reducing exposure to surrounding normal tissues. Early evidence suggests a more favourable toxicity profile, particularly regarding structures involved in salivary function and swallowing. However, data specifically demonstrating improved nutritional outcomes or superior treatment tolerance among nutritionally vulnerable patients remain limited (47, 48).

Overall, malnutrition and sarcopenia should be regarded as clinically relevant predictors of poorer treatment tolerance in patients with HNC, rather than established causal factors. These conditions are consistently associated with greater vulnerability to treatment-related toxicity, increased supportive-care requirements, treatment interruption, and less favourable clinical outcomes. Treatment interruptions and unplanned dose reductions may compromise treatment intensity and consequently be associated with poorer oncologic outcomes, reinforcing the importance of early nutritional identification and intervention (8). Consequently, systematic nutritional screening, comprehensive nutritional assessment, and early supportive interventions should be integrated throughout the oncologic pathway to optimize treatment delivery and improve patient outcomes (4, 15, 18).

### 3.3 Sarcopenia and body composition as prognostic biomarkers

Sarcopenia and other adverse body-composition phenotypes have emerged as clinically relevant prognostic biomarkers in patients with HNC (12, 30, 44). Beyond reflecting nutritional status, alterations in skeletal muscle quantity and quality may provide important information regarding physiologic reserve, functional capacity, resilience to treatment-related stressors, and overall prognosis (28). Consequently, body-composition assessment offers clinically meaningful information that cannot be captured by conventional anthropometric parameters such as body weight or BMI, particularly in patients with sarcopenic obesity, in whom substantial muscle depletion may coexist with preserved or excess adiposity and therefore remain undetected by traditional nutritional assessment methods (14, 29).

Although many oncological studies have used the term *radiologically defined sarcopenia* to describe low skeletal muscle mass identified on CT imaging, this approach should not be considered equivalent to the clinical diagnosis of sarcopenia according to current consensus definitions, which also incorporate muscle strength and, where appropriate, physical performance. Despite this distinction, CT-derived muscle depletion has consistently demonstrated prognostic value in patients with HNC.

A growing body of evidence supports the prognostic significance of radiologically defined sarcopenia across different HNC populations and treatment modalities. In a meta-analysis including 3,233 patients with HNC, Takenaka et al. (44) demonstrated that radiologically defined sarcopenia was associated with significantly poorer overall survival across both surgical and radiotherapy cohorts, highlighting the prognostic relevance of skeletal muscle depletion irrespective of treatment modality. Similarly, Findlay et al. (12) reported that pre-treatment radiologically defined sarcopenia was independently associated with significantly poorer overall survival in patients undergoing curative-intent radiotherapy for HNC. More recently, van Heusden et al. (30) performed a comprehensive systematic review and multilevel meta-analysis including more than 14,000 patients and confirmed that radiologically defined sarcopenia was consistently associated with reduced overall survival, increased treatment-related complications, and poorer clinical outcomes across different treatment settings. Collectively, these findings support considering the incorporation of body-composition assessment into routine risk stratification and prognostic evaluation of patients with HNC.

The prognostic significance of body composition is not limited to survival alone. Several studies have demonstrated associations between skeletal muscle depletion and increased treatment-related toxicity, postoperative complications, prolonged hospitalization, impaired functional recovery, and reduced treatment completion rates (8, 30, 44–46). Jung et al. demonstrated that unfavourable body-composition profiles independently predicted recurrence and mortality in patients with advanced-stage HNC, even after adjustment for established prognostic factors (13). Likewise, Stone et al. identified preoperative radiologically defined sarcopenia as an independent predictor of poorer long-term survival following major head and neck cancer surgery, further supporting the prognostic value of skeletal muscle assessment in this population (49). From a biological perspective, these associations likely reflect reduced physiologic reserve and a diminished capacity to tolerate the metabolic, inflammatory, and functional stress imposed by both the tumour and its treatment. Taken together, these findings suggest that body-composition assessment provides clinically relevant prognostic information beyond traditional clinicopathological variables and may contribute to improved risk stratification in patients with HNC.

Another emerging area of interest is muscle quality. Increasing evidence suggests that myosteatosis, characterized by fatty infiltration and reduced radiodensity of skeletal muscle, may provide prognostic information beyond muscle quantity alone. In a prospective cohort study, Ahern et al. reported that post-treatment radiologically defined sarcopenia was associated with poorer long-term survival and that this adverse prognostic effect appeared particularly evident in patients with concomitant myosteatosis (50). These observations support the growing concept that muscle quantity and muscle quality should be regarded as complementary rather than competing biomarkers.

Despite the consistency of these associations, several methodological challenges remain. Considerable heterogeneity persists across studies regarding the definitions of radiologically defined sarcopenia and clinically defined sarcopenia, imaging-based assessment methods, anatomical landmarks used for muscle quantification, and cut-off values used to define low skeletal muscle mass (51, 52). These differences may contribute to variability in prevalence estimates, patient classification, and prognostic thresholds across studies. Furthermore, most available evidence is derived from retrospective observational cohorts, which limits causal inference and may be influenced by residual confounding. Future prospective studies using standardized assessment methods and consensus diagnostic criteria are needed to further clarify the prognostic role of body-composition abnormalities in patients with HNC.

### 3.4 Morphofunctional assessment in clinical practice

#### 3.4.1 Body composition assessment

**Figure 2:**
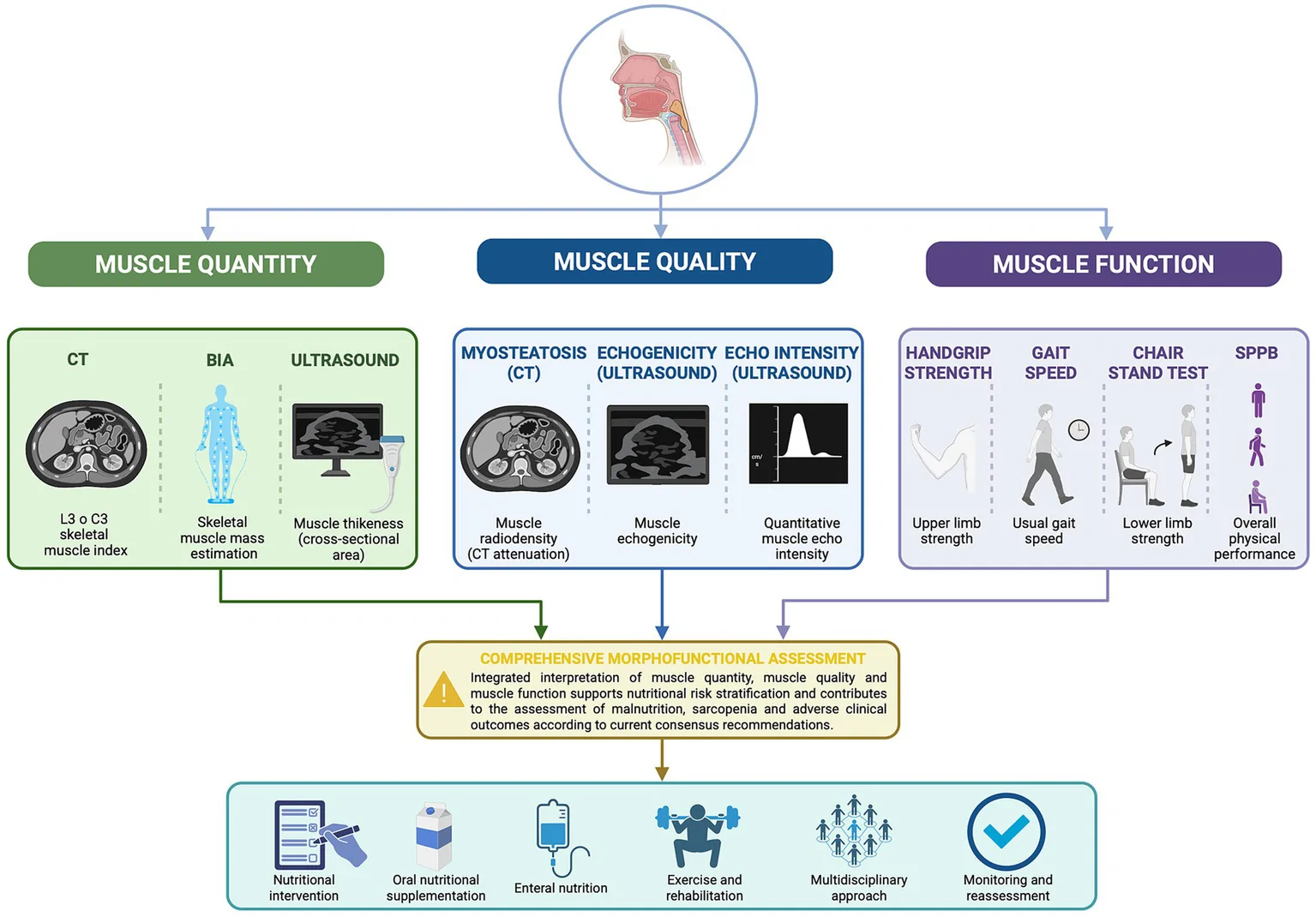
Morphofunctional assessment in patients with head and neck cancer. Created in BioRender. Cebrián Vázquez, A. (2026), https://BioRender.com/05wxlu2.

Morphofunctional assessment has emerged as a fundamental component of nutritional evaluation in patients with HNC. Unlike conventional anthropometric parameters such as body weight or BMI, morphofunctional assessment provides a more comprehensive characterization of nutritional status by integrating body-composition analysis with measures of muscle strength and physical performance. This approach is particularly relevant in HNC, where clinically significant skeletal muscle depletion may occur despite apparently preserved body weight, especially in patients with sarcopenic obesity (14). Contemporary malnutrition frameworks, including the Global Leadership Initiative on Malnutrition (GLIM), recommend a two-step approach consisting of initial nutritional risk screening followed by the diagnosis of malnutrition based on the presence of at least one phenotypic criterion and one etiologic criterion. Within this framework, reduced muscle mass is recognized as one of the phenotypic criteria, highlighting the importance of incorporating muscle assessment into routine nutritional evaluation (15, 53). Although malnutrition, sarcopenia, and cachexia frequently coexist in patients with HNC, they represent distinct clinical entities with different diagnostic criteria and should not be used interchangeably. Additional nutritional assessment tools, including the Geriatric Nutritional Risk Index (GNRI) and the Prognostic Nutritional Index (PNI), have also been investigated in patients with HNC. These indices, which are based on anthropometric and laboratory parameters, have shown prognostic value and may complement comprehensive nutritional assessment, although they do not directly evaluate muscle mass or physical function (54, 55). The principal components of morphofunctional assessment and their integration into risk stratification and personalized management are summarized in Figure 2.

Several techniques are available for body-composition assessment. Bioelectrical impedance analysis (BIA) is widely used in clinical practice because it is inexpensive, rapid, non-invasive, portable, and easily repeatable (53). In addition to providing estimates of skeletal muscle mass, fat-free mass, body cell mass, and hydration status, BIA-derived phase angle has emerged as a promising marker of cellular integrity and nutritional status, with growing evidence supporting its prognostic value in oncology populations (56). Nevertheless, the accuracy of BIA may be influenced by hydration abnormalities, edema, and other clinical factors, which should be carefully considered when interpreting results (53).

Computed tomography (CT) remains one of the reference methods for body-composition assessment in oncology and is currently the most widely used imaging modality for opportunistic evaluation of skeletal muscle mass in patients with HNC (57, 58). Quantification of skeletal muscle cross-sectional area at the third lumbar vertebra (L3) has traditionally been considered the reference standard for estimating whole-body muscle mass. Because abdominal imaging is not routinely available in many patients with HNC, alternative approaches based on cervical imaging have been developed. Assessment of skeletal muscle at the third cervical vertebra (C3) has demonstrated good correlation with L3-derived measurements and enables the identification of low skeletal muscle mass using imaging studies routinely obtained during cancer staging and follow-up (51, 59). Beyond muscle quantity, CT-based analyses also allow evaluation of muscle quality through measures of radiodensity, facilitating the identification of myosteatosis and other adverse body-composition phenotypes that have been associated with poorer clinical outcomes (50, 58). Dual-energy X-ray absorptiometry (DXA) is also considered a reference technique for the assessment of body composition and appendicular lean mass and is widely used in sarcopenia research. However, its routine application in HNC is limited by the need for dedicated examinations, whereas CT-based assessment can be performed opportunistically using imaging studies already acquired for oncologic management (53, 57).

#### 3.4.2 Muscle function assessment

Assessment of muscle function constitutes an equally important component of morphofunctional evaluation. Handgrip strength is one of the most widely used functional measures because of its simplicity, reproducibility, and strong association with clinically relevant outcomes. Additional measures of physical performance include gait speed, chair-stand tests, and composite instruments such as the Short Physical Performance Battery (SPPB), which incorporates balance, gait speed, and chair-stand performance. According to the European Working Group on Sarcopenia in Older People (EWGSOP2), low muscle strength represents the primary criterion for identifying probable sarcopenia, whereas reduced muscle quantity or quality confirms the diagnosis and impaired physical performance indicates severe sarcopenia (28). Together, these measures may identify impairments that are not fully captured by muscle-mass assessment alone and contribute to a broader evaluation of patient function and overall physiologic reserve.

#### 3.4.3 Muscle ultrasound

More recently, muscle ultrasound has emerged as a promising bedside tool for morphofunctional assessment. Ultrasound is non-invasive, radiation-free, portable, relatively inexpensive, and well suited for repeated longitudinal evaluations. The quadriceps muscle group, particularly the rectus femoris, has been extensively studied in geriatric and general oncology populations, allowing assessment of both muscle quantity (thickness and cross-sectional area) and muscle quality through echogenicity and echo intensity, parameters associated with fatty infiltration, fibrosis, and structural muscle deterioration (17). Furthermore, ultrasound may facilitate longitudinal monitoring of changes in muscle status during oncologic treatment and the evaluation of responses to nutritional and exercise interventions. Recent prospective studies in patients with HNC suggest that ultrasound-derived muscle parameters are associated with nutritional status, sarcopenia, and survival outcomes. Although these findings are encouraging, the available evidence remains limited and further validation is required before widespread clinical implementation (60).

Interest has also expanded toward the assessment of craniofacial muscles, particularly the masseter muscle, as a potential biomarker of nutritional and functional status in HNC. Given the central role of the masseter muscle in mastication and oral intake, alterations in this muscle may reflect both disease burden and treatment-related functional impairment. Emerging evidence suggests that reduced masseter muscle thickness is associated with cancer cachexia and adverse nutritional status, while masseter muscle measurements have shown potential prognostic value for survival outcomes in patients with HNC (61, 62). Taking together, current evidence suggests that muscle ultrasound may represent a valuable complementary tool for morphofunctional assessment in HNC. Nevertheless, its implementation requires careful standardization, as measurements remain operator dependent and may be influenced by transducer pressure, patient positioning, anatomical acquisition site, and ultrasound equipment settings. Further prospective studies are required to establish disease-specific reference values, diagnostic cut-off points, echogenicity standardization, and inter-observer reproducibility before routine implementation in clinical practice.

Overall, morphofunctional assessment provides a multidimensional characterization of nutritional and functional status that extends beyond conventional anthropometric evaluation. By integrating body-composition analysis with measures of muscle strength and physical performance, this approach may facilitate earlier identification of vulnerable patients, improve risk stratification, and support individualized nutritional, rehabilitative, and supportive-care strategies throughout the oncologic trajectory (53, 58).

### 3.5 Nutritional and supportive interventions

#### 3.5.1 Nutritional counselling and nutritional support

The high prevalence of malnutrition and sarcopenia among patients with HNC, together with their association with treatment-related toxicity, impaired treatment tolerance, and poorer clinical outcomes, underscores the importance of early nutritional intervention. Nutritional care should be initiated at the time of cancer diagnosis and integrated throughout the cancer care continuum, running in parallel with antineoplastic treatment rather than being reserved for patients with established weight loss or advanced nutritional deterioration (4, 5). Current guidelines recommend systematic nutritional screening followed by comprehensive nutritional assessment and individualized nutritional support according to nutritional risk, disease burden, treatment modality, and functional status (4). In patients with HNC, specialist dietitians play a central role within the multidisciplinary team, contributing to the prevention, early identification, and management of disease- and treatment-related malnutrition throughout the course of treatment (27).

Optimal nutritional management in patients with HNC also requires careful attention to oral health and dental care. Poor dentition, impaired masticatory function, periodontal disease, odontogenic pain, ill-fitting dental prostheses, and oral infections may substantially compromise oral intake and contribute to malnutrition. In addition, treatment-related complications such as mucositis, xerostomia, tooth extractions, and osteoradionecrosis can further impair chewing, swallowing, and nutritional status. Early dental assessment, preventive oral care, and oral rehabilitation should therefore be integrated into supportive care to preserve oral function, optimize nutritional intake, and improve treatment tolerance (63).

Within this multidisciplinary approach, individualized nutritional counselling remains the cornerstone of nutritional management in HNC. Given the considerable heterogeneity in tumour site, disease stage, treatment modality, and nutrition-impact symptoms, dietary recommendations should be tailored to each patient’s clinical and functional status. Nutritional counselling should address not only individualized energy and protein requirements but also the progressive functional limitations associated with dysphagia, odynophagia, xerostomia, mucositis, and taste alterations, which frequently compromise oral intake during treatment (4, 27). Regular follow-up by specialist dietitians facilitates the early identification of nutritional deterioration, timely adjustment of nutritional interventions, and proactive management of treatment-related symptoms, thereby contributing to the maintenance of nutritional status throughout treatment.

When dietary counselling alone is insufficient to meet nutritional requirements, oral nutritional supplements (ONS) represent the recommended first-line strategy for patients who retain a functional gastrointestinal tract. High-energy, high-protein formulations may improve energy and protein intake and help attenuate treatment-related weight loss, particularly when introduced early as part of an individualized nutritional programme that includes ongoing dietary counselling (4, 27). However, ONS should not be considered an isolated intervention but rather part of a comprehensive nutritional care pathway that includes regular reassessment of nutritional status and adjustment of nutritional support according to the patient’s clinical course, treatment-related toxicities, and changes in functional capacity.

For patients with severe dysphagia, marked weight loss, or persistent inability to meet nutritional requirements through oral intake, enteral nutrition becomes an essential component of supportive care. Both nasogastric tubes (NGT) and percutaneous endoscopic gastrostomy (PEG) are widely used during radiotherapy and chemoradiotherapy, although the optimal timing of PEG placement remains controversial. Current evidence suggests that prophylactic PEG may reduce treatment-related weight loss and the need for unplanned nutritional interventions in selected high-risk patients, whereas a reactive approach avoids prophylactic tube placement in individuals who ultimately maintain adequate oral intake (64, 65). Rather than supporting a universal strategy, recent systematic reviews emphasize individualized decision-making based on baseline nutritional status, swallowing function, anticipated treatment-related toxicity, tumour characteristics, and patient preferences (4, 64). Importantly, enteral nutrition should be regarded as a supportive measure to maintain adequate nutrition while preserving oral intake and swallowing function whenever feasible, highlighting the importance of close collaboration between nutrition specialists, speech and language therapists, and the multidisciplinary oncology team.

#### 3.5.2 Targeted nutritional strategies

Because cancer cachexia is driven by complex metabolic and inflammatory mechanisms, nutritional support alone is often insufficient to completely prevent the progressive loss of skeletal muscle. Consequently, several targeted nutritional strategies have been investigated as adjuncts to conventional nutritional support. Long-chain omega-3 fatty acids have been proposed to attenuate systemic inflammation and muscle wasting, whereas anabolic nutritional substrates such as *β*-hydroxy-β-methylbutyrate (HMB) and leucine may contribute to the preservation of muscle protein synthesis. Although these approaches are supported by a sound biological rationale and have shown encouraging results in selected studies, current evidence remains inconsistent and insufficient to recommend their routine use in patients with HNC. Therefore, these interventions should be considered complementary to, rather than substitutes for, individualized nutritional counselling and comprehensive multimodal supportive care (4, 66).

#### 3.5.3 Exercise and prehabilitation

Increasing attention has focused on multimodal interventions aimed at preserving skeletal muscle mass, muscle function, and overall physiologic reserve throughout the cancer trajectory. Nutritional support remains the cornerstone of management, but growing interest has centred on combining individualized nutritional care with structured exercise programmes, particularly resistance training, to attenuate treatment-related functional decline and enhance recovery. Current evidence, including systematic reviews of randomized controlled trials, suggests that multimodal interventions integrating nutritional support and exercise may improve physical function, muscle strength, and health-related quality of life in patients with HNC. However, evidence demonstrating improvements in treatment tolerance, treatment-related toxicity, oncologic outcomes, or survival remains limited. Further high-quality prospective studies are needed to determine their impact on long-term oncologic and functional outcomes (67–69).

Prehabilitation has emerged as a proactive, multimodal strategy aimed at optimizing patients’ functional, nutritional, and psychological status between cancer diagnosis and treatment initiation, with the goal of improving treatment tolerance, reducing complications, and enhancing recovery (70, 71). In oncology, prehabilitation typically integrates nutritional optimization, structured exercise, psychological support, and the management of disease-specific functional impairments within a multidisciplinary framework. Although evidence specifically addressing HNC remains limited, recent studies suggest that multimodal prehabilitation programmes are feasible, well accepted by patients, and may improve functional outcomes and treatment preparedness (72–74). Nevertheless, the available evidence is still largely based on feasibility studies with relatively small sample sizes and heterogeneous interventions, and further prospective research is required to better define the role of prehabilitation within standard multidisciplinary care for patients with HNC (72–74).

Taken together, current evidence supports a shift from reactive nutritional support towards proactive, multimodal supportive care in patients with HNC. Early nutritional intervention, individualized dietary counselling, appropriate nutritional supplementation, structured exercise, and prehabilitation should be regarded as complementary rather than isolated strategies within multidisciplinary cancer care. Although the evidence supporting several of these interventions continues to evolve, their integration provides a rational multidisciplinary framework to support nutritional status, physical function, and supportive care throughout the oncologic trajectory, while further randomized clinical trials are needed to establish their effects on treatment tolerance, treatment-related toxicity, and survival (4, 5).

The principal evidence supporting the clinical relevance of malnutrition, body composition abnormalities, morphofunctional assessment, and supportive interventions in HNC is summarized in Table 1.

**Table 1:** Summary of the current evidence on malnutrition, body composition, morphofunctional assessment, and supportive interventions in patients with head and neck cancer.

Theme	Representative evidence	Evidence base	Overall evidence
Malnutrition and treatment tolerance	(8, 10, 11)	Systematic reviews + cohort studies	Malnutrition is consistently associated with poorer treatment tolerance, increased treatment-related toxicity during chemoradiotherapy, and adverse postoperative outcomes, including higher complication rates and prolonged hospital stay, emphasizing the importance of early nutritional assessment and intervention.
Nutrition impact symptoms	(23, 27, 31, 35)	Systematic reviews + narrative reviews + expert consensus	Dysphagia, mucositis, xerostomia and dysgeusia impair oral intake, contribute to nutritional deterioration and increase the need for nutritional support during treatment.
Sarcopenia as a prognostic biomarker	(12, 13, 30, 44, 49, 50)	Meta-analyses + systematic reviews + cohort studies	Sarcopenia and low skeletal muscle mass are consistently associated with poorer overall survival, reduced disease-free survival, and adverse clinical outcomes, with growing evidence supporting their association with increased treatment-related toxicity and poorer treatment tolerance.
Sarcopenia and surgical outcomes	(45, 46)	Systematic reviews and meta-analyses	Low skeletal muscle mass is consistently associated with an increased risk of postoperative complications following head and neck cancer surgery, particularly in patients undergoing free-flap reconstruction.
CT-based body composition assessment	(51, 52, 59)	Validation studies	CT assessment at the third cervical vertebra (C3) is a validated surrogate for estimating skeletal muscle mass in patients with head and neck cancer, facilitating opportunistic body composition assessment without additional imaging.
Muscle ultrasound	Perkisas et al. (17), SARCUS; (60–62)	Expert consensus + observational studies	Muscle ultrasound is a promising complementary bedside tool for the assessment of muscle quantity and quality. Emerging evidence suggests a potential role in risk stratification and prognostic assessment in patients with head and neck cancer. However, further standardization of acquisition protocols, including patient positioning, transducer pressure, ultrasound settings, operator training, disease-specific reference values, diagnostic cut-off points, and inter-observer reproducibility, is required before routine implementation in clinical practice.
Comprehensive nutritional and muscle assessment	Cederholm et al. (15), GLIM Consensus; Muscaritoli et al. (4), ESPEN Practical Guideline; Barazzoni et al. (53), GLIM Muscle Mass Guidance; Cruz-Jentoft et al. (28), EWGSOP2 Consensus	International consensus statements + clinical practice guidelines	Current consensus recommendations support a comprehensive assessment integrating nutritional screening, body composition and muscle function to enable early identification of patients at nutritional risk and guide individualized nutritional management.
Nutritional support and enteral nutrition	Muscaritoli et al. (4), ESPEN practical guideline; (5, 27, 64, 65)	Clinical practice guidelines + systematic reviews + expert recommendations	Early nutritional counselling, individualized dietary intervention and appropriate enteral nutrition are essential components of nutritional care throughout treatment, although the optimal timing of prophylactic feeding tube placement remains under debate.
Exercise and prehabilitation	(67–71)	Narrative reviews + systematic reviews of randomized controlled trials + meta-analyses	Exercise and multimodal prehabilitation are associated with improvements in physical function, quality of life and functional recovery, although evidence for improvements in treatment tolerance and oncological outcomes is still limited.

### 3.6 Future perspectives and research gaps

Despite the growing recognition of malnutrition, sarcopenia, and adverse body-composition phenotypes as clinically relevant predictors of treatment tolerance and outcomes in patients with HNC, their integration into routine oncologic care remains limited. Current guidelines recommend systematic nutritional screening and comprehensive nutritional assessment (4, 15), yet the routine assessment of muscle mass, muscle quality, and physical function remains constrained by methodological heterogeneity, lack of standardized cut-off values, and incomplete implementation within standard supportive oncology pathways (15, 17, 30). To date, most available studies, particularly those evaluating radiologically defined sarcopenia, have relied on single baseline assessments, providing only a static representation of nutritional and functional status. Preliminary longitudinal evidence suggests that changes in body composition during treatment may have greater prognostic relevance than baseline measurements alone, reinforcing the need for serial morphofunctional assessment throughout the oncologic trajectory (30, 75). Future research should therefore focus on integrating longitudinal morphofunctional assessment into clinical practice, moving beyond the identification of prognostic biomarkers towards dynamic monitoring of muscle quantity, muscle quality, and physical function. Such an approach may improve risk stratification, facilitate earlier therapeutic interventions, and ultimately support more personalized nutritional and rehabilitative care.

The increasing availability of artificial intelligence (AI), radiomics, and automated image analysis is expected to facilitate the integration of body-composition assessment into routine oncologic care (76, 77). Automated extraction of body-composition parameters from routinely acquired CT imaging could enable efficient and reproducible identification of low skeletal muscle mass and radiologically defined sarcopenia, myosteatosis, and other adverse body-composition phenotypes without increasing clinical workload (77). More importantly, future predictive models should move beyond isolated imaging metrics by integrating imaging-derived biomarkers with morphofunctional, nutritional, inflammatory, and clinical variables to provide comprehensive decision-support tools capable of predicting treatment toxicity, treatment tolerance, postoperative complications, and survival (76, 77). Rather than serving solely as diagnostic tools, these approaches may facilitate more personalized supportive care by identifying patients most likely to benefit from early nutritional intervention, structured exercise programmes, and multimodal prehabilitation.

Muscle ultrasound has emerged as a promising tool for morphofunctional assessment. Its bedside applicability, absence of ionizing radiation, low cost, and ability to provide repeated evaluations make it particularly attractive for longitudinal monitoring in patients with HNC (17, 62). Beyond the assessment of muscle quantity, ultrasound can provide complementary information on muscle quality through echogenicity and other structural characteristics, potentially enabling earlier detection of treatment-related muscle deterioration. Growing interest has also focused on craniofacial muscles, particularly the masseter and temporalis muscles, as clinically relevant biomarkers of nutritional and functional status (61, 62). Nevertheless, prospective multicentre studies are still required to standardize acquisition protocols, establish population-specific reference values and diagnostic cut-off points, and clarify the added clinical value of ultrasound-derived morphofunctional parameters for risk stratification, treatment decision-making, and longitudinal monitoring before widespread implementation in routine clinical practice.

Ultimately, the next generation of prospective research should determine whether morphofunctional assessment can evolve from a prognostic tool into a therapeutic decision-support strategy. Prospective randomized studies integrating longitudinal morphofunctional assessment with individualized nutritional interventions, structured exercise programmes, and multimodal prehabilitation are needed to establish whether this approach improves treatment tolerance, completion of oncologic therapy, postoperative recovery, quality of life, and survival. The transition from simple risk stratification towards personalized, risk-adapted supportive care may represent an important step towards precision supportive oncology in patients with HNC.

### 3.7 Limitations

This review has several limitations inherent to its narrative design. A systematic literature search and formal assessment of study quality or risk of bias were not performed. In addition, substantial heterogeneity exists across the available literature regarding the definitions of clinically defined and radiologically defined sarcopenia, body-composition assessment methods, muscle function assessment, and the clinical outcomes evaluated, limiting direct comparison between studies. Variability in imaging techniques, anatomical landmarks, and cut-off values used to define low skeletal muscle mass further complicates the interpretation and generalizability of findings. Furthermore, much of the available evidence is derived from retrospective observational studies, limiting causal inference. Reported associations between malnutrition, sarcopenia, body-composition abnormalities, and clinical outcomes may be influenced by residual confounding related to disease stage, systemic inflammation, frailty, comorbidities, treatment intensity, and other patient- and disease-related factors. In addition, reverse causality cannot be excluded, as more advanced disease may simultaneously contribute to muscle depletion, poorer nutritional status, and worse clinical outcomes. Although many studies have adjusted for important clinical variables, the extent of adjustment varies considerably, and therefore an observed association should not necessarily be interpreted as independent prognostic value. Prospective longitudinal studies evaluating dynamic changes in morphofunctional parameters, together with adequately adjusted prospective cohorts and randomized intervention studies, are needed to strengthen prognostic inference and clarify potential causal relationships. Nevertheless, by integrating recent evidence from nutritional, oncologic, and morphofunctional research, this review provides a contemporary overview of current knowledge and highlights key areas for future research and clinical implementation in patients with head and neck cancer.

## 4 Conclusion

Malnutrition, sarcopenia, and adverse body-composition phenotypes are highly prevalent in patients with head and neck cancer and are consistently associated with poorer treatment tolerance, increased treatment-related toxicity, and less favourable clinical outcomes. Growing evidence highlights the clinical relevance of morphofunctional assessment, demonstrating that the evaluation of muscle quantity, muscle quality, and physical function provides valuable information beyond conventional anthropometric measures alone.

In this context, the integration of morphofunctional assessment into routine clinical practice has the potential to facilitate earlier identification of patients at nutritional risk and support more individualized nutritional and rehabilitative interventions throughout the oncologic trajectory. Beyond improving risk stratification, morphofunctional assessment has the potential to evolve into a clinical tool capable of guiding personalized supportive care and monitoring response to multimodal interventions over time. Continued efforts to standardize assessment methods, validate emerging tools such as muscle ultrasound, and establish longitudinal monitoring strategies will be essential to facilitate the translation of these advances into routine care and ultimately contribute to improved outcomes for patients with head and neck cancer.
